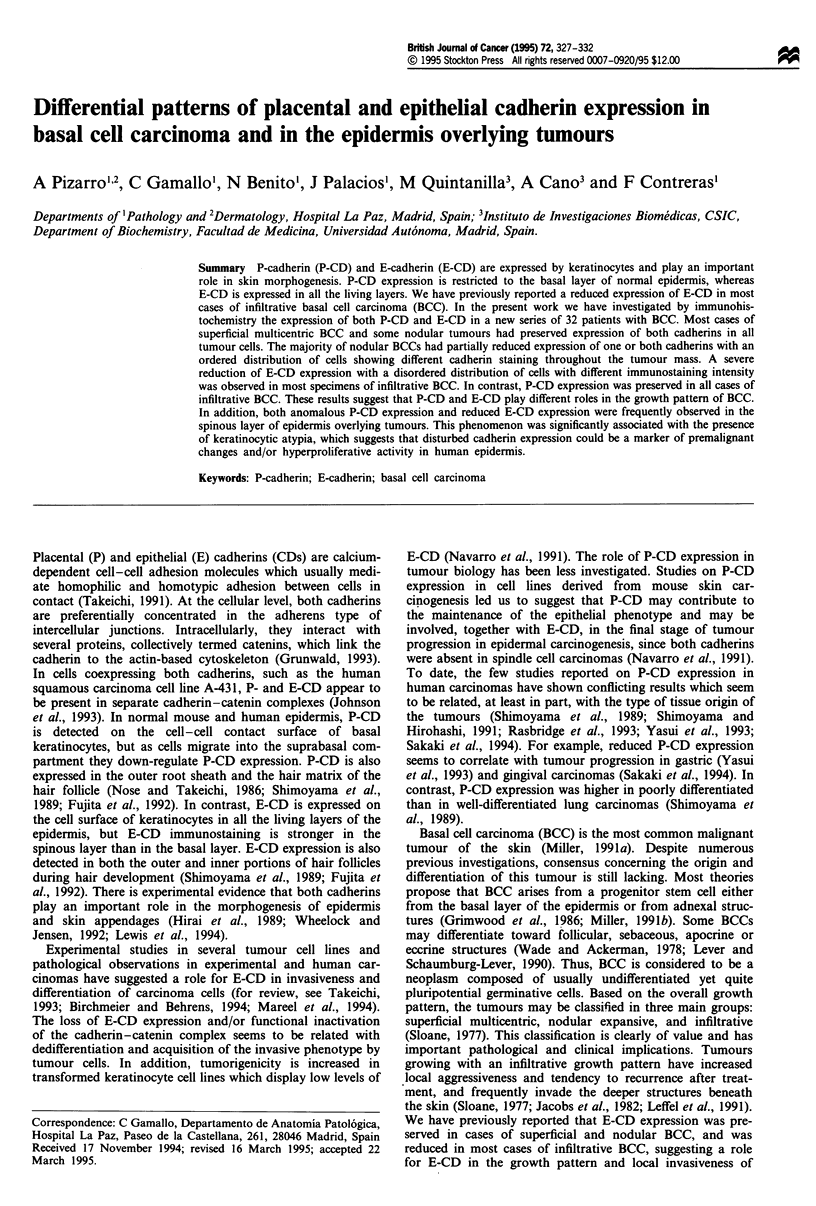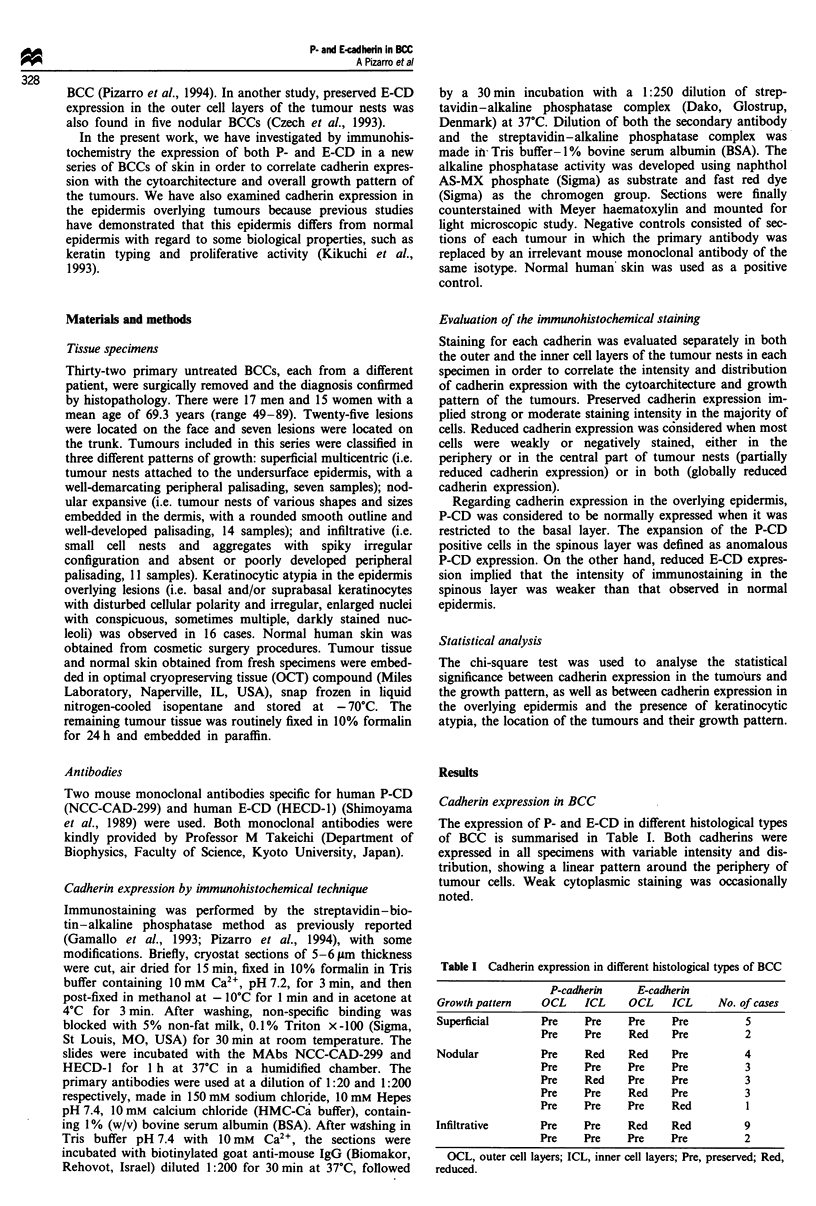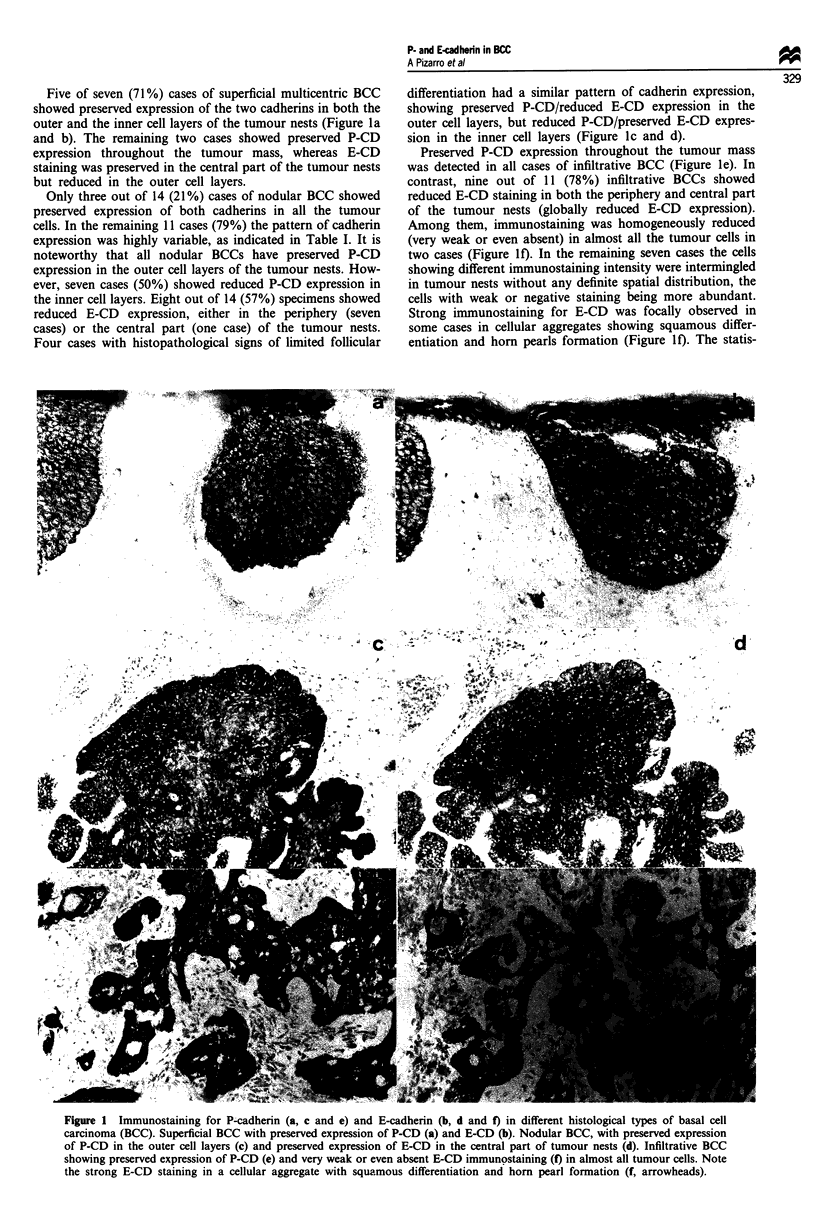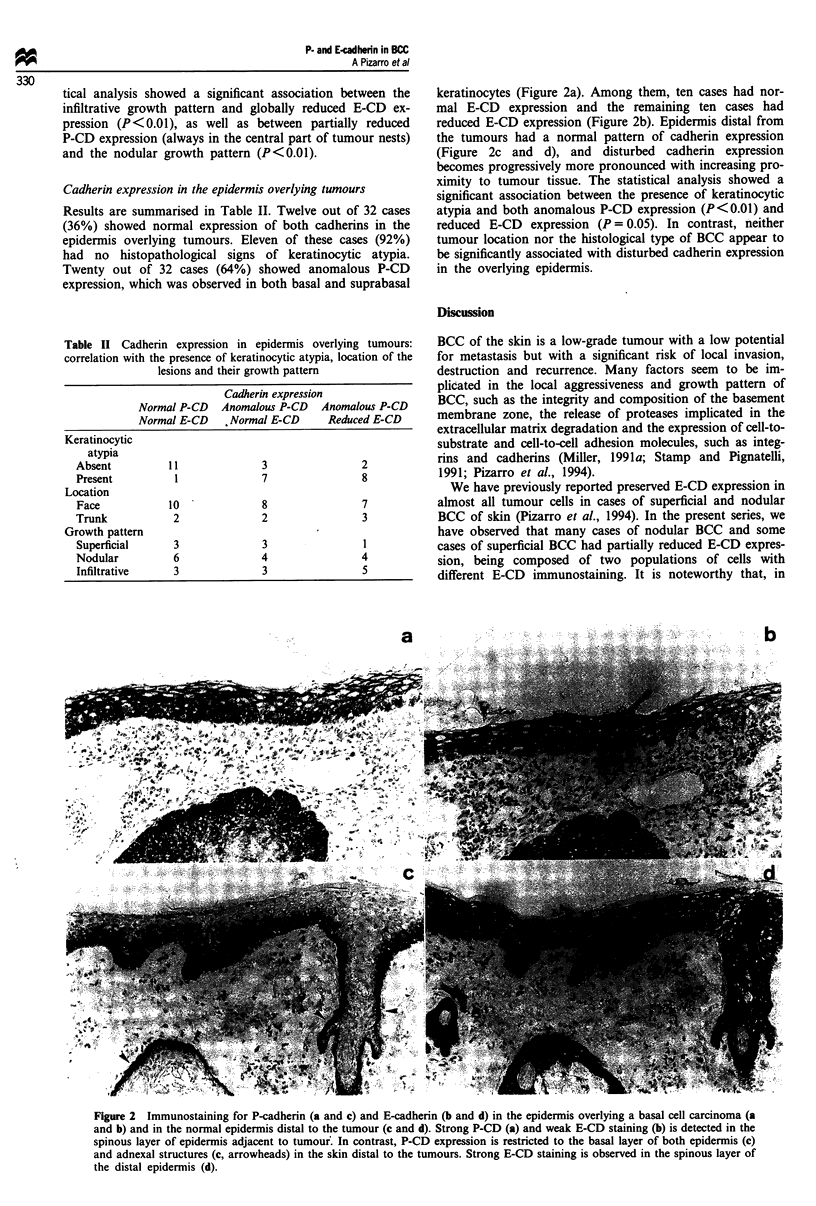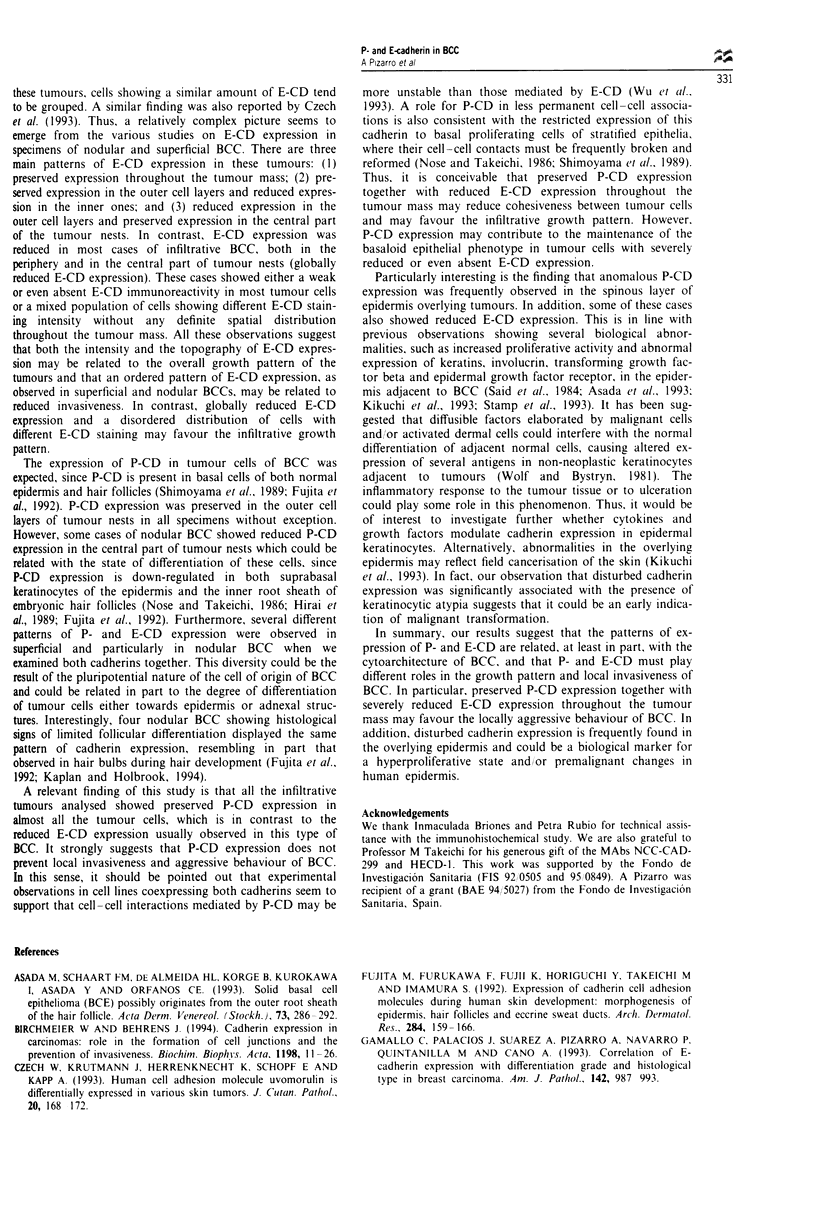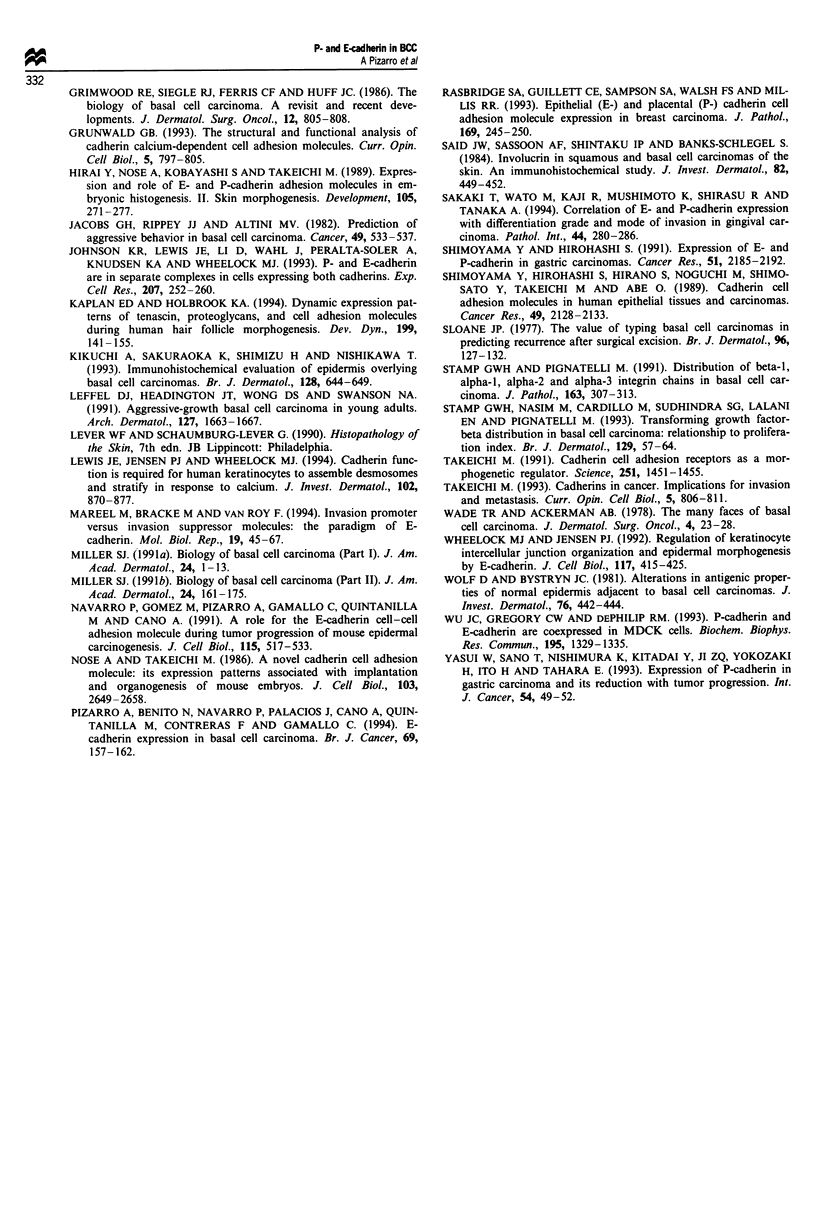# Differential patterns of placental and epithelial cadherin expression in basal cell carcinoma and in the epidermis overlying tumours.

**DOI:** 10.1038/bjc.1995.333

**Published:** 1995-08

**Authors:** A. Pizarro, C. Gamallo, N. Benito, J. Palacios, M. Quintanilla, A. Cano, F. Contreras

**Affiliations:** Department of Pathology, Hospital La Paz, Madrid, Spain.

## Abstract

**Images:**


**British Journal of Cancer (1995) 72, 327–332**
© 1995 Stockton Press All rights reserved 0007–0920/95 $12.00

### Differential patterns of placental and epithelial cadherin expression in basal cell carcinoma and in the epidermis overlying tumours

A Pizarro[1,2], C Gamallo[1], N Benito[1], J Palacios[1], M Quintanilla[3], A Cano[3] and F Contreras[1]

*Departments of [1]Pathology and [2]Dermatology, Hospital La Paz, Madrid, Spain; [3]Instituto de Investigaciones Biomédicas, CSIC, Department of Biochemistry, Facultad de Medicina, Universidad Autónoma, Madrid, Spain.*

**Summary** P-cadherin (P-CD) and E-cadherin (E-CD) are expressed by keratinocytes and play an important role in skin morphogenesis. P-CD expression is restricted to the basal layer of normal epidermis, whereas E-CD is expressed in all the living layers. We have previously reported a reduced expression of E-CD in most cases of infiltrative basal cell carcinoma (BCC). In the present work we have investigated by immunohistochemistry the expression of both P-CD and E-CD in a new series of 32 patients with BCC. Most cases of superficial multicentric BCC and some nodular tumours had preserved expression of both cadherins in all tumour cells. The majority of nodular BCCs had partially reduced expression of one or both cadherins with an ordered distribution of cells showing different cadherin staining throughout the tumour mass. A severe reduction of E-CD expression with a disordered distribution of cells with different immunostaining intensity was observed in most specimens of infiltrative BCC. In contrast, P-CD expression was preserved in all cases of infiltrative BCC. These results suggest that P-CD and E-CD play different roles in the growth pattern of BCC. In addition, both anomalous P-CD expression and reduced E-CD expression were frequently observed in the spinous layer of epidermis overlying tumours. This phenomenon was significantly associated with the presence of keratinocytic atypia, which suggests that disturbed cadherin expression could be a marker of premalignant changes and/or hyperproliferative activity in human epidermis.

**Keywords**: P-cadherin; E-cadherin; basal cell carcinoma

Placental (P) and epithelial (E) cadherins (CDs) are calcium-dependent cell–cell adhesion molecules which usually mediate homophilic and homotypic adhesion between cells in contact (Takeichi, 1991). At the cellular level, both cadherins are preferentially concentrated in the adherens type of intercellular junctions. Intracellularly, they interact with several proteins, collectively termed catenins, which link the cadherin to the actin-based cytoskeleton (Grunwald, 1993). In cells coexpressing both cadherins, such as the human squamous carcinoma cell line A-431, P- and E-CD appear to be present in separate cadherin–catenin complexes (Johnson *et al.*, 1993). In normal mouse and human epidermis, P-CD is detected on the cell–cell contact surface of basal keratinocytes, but as cells migrate into the suprabasal compartment they down-regulate P-CD expression. P-CD is also expressed in the outer root sheath and the hair matrix of the hair follicle (Nose and Takeichi, 1986; Shimoyama *et al.*, 1989; Fujita *et al.*, 1992). In contrast, E-CD is expressed on the cell surface of keratinocytes in all the living layers of the epidermis, but E-CD immunostaining is stronger in the spinous layer than in the basal layer. E-CD expression is also detected in both the outer and inner portions of hair follicles during hair development (Shimoyama *et al.*, 1989; Fujita *et al.*, 1992). There is experimental evidence that both cadherins play an important role in the morphogenesis of epidermis and skin appendages (Hirai *et al.*, 1989; Wheelock and Jensen, 1992; Lewis *et al.*, 1994).

Experimental studies in several tumour cell lines and pathological observations in experimental and human carcinomas have suggested a role for E-CD in invasiveness and differentiation of carcinoma cells (for review, see Takeichi, 1993; Birchmeier and Behrens, 1994; Mareel *et al.*, 1994). The loss of E-CD expression and/or functional inactivation of the cadherin–catenin complex seems to be related with dedifferentiation and acquisition of the invasive phenotype by tumour cells. In addition, tumorigenicity is increased in transformed keratinocyte cell lines which display low levels of E-CD (Navarro *et al.*, 1991). The role of P-CD expression in tumour biology has been less investigated. Studies on P-CD expression in cell lines derived from mouse skin carcinogenesis led us to suggest that P-CD may contribute to the maintenance of the epithelial phenotype and may be involved, together with E-CD, in the final stage of tumour progression in epidermal carcinogenesis, since both cadherins were absent in spindle cell carcinomas (Navarro *et al.*, 1991). To date, the few studies reported on P-CD expression in human carcinomas have shown conflicting results which seem to be related, at least in part, with the type of tissue origin of the tumours (Shimoyama *et al.*, 1989; Shimoyama and Hirohashi, 1991; Rasbridge *et al.*, 1993; Yasui *et al.*, 1993; Sakaki *et al.*, 1994). For example, reduced P-CD expression seems to correlate with tumour progression in gastric (Yasui *et al.*, 1993) and gingival carcinomas (Sakaki *et al.*, 1994). In contrast, P-CD expression was higher in poorly differentiated than in well-differentiated lung carcinomas (Shimoyama *et al.*, 1989).

Basal cell carcinoma (BCC) is the most common malignant tumour of the skin (Miller, 1991*a*). Despite numerous previous investigations, consensus concerning the origin and differentiation of this tumour is still lacking. Most theories propose that BCC arises from a progenitor stem cell either from the basal layer of the epidermis or from adnexal structures (Grimwood *et al.*, 1986; Miller, 1991*b*). Some BCCs may differentiate toward follicular, sebaceous, apocrine or eccrine structures (Wade and Ackerman, 1978; Lever and Schaumburg-Lever, 1990). Thus, BCC is considered to be a neoplasm composed of usually undifferentiated yet quite pluripotential germinative cells. Based on the overall growth pattern, the tumours may be classified in three main groups: superficial multicentric, nodular expansive, and infiltrative (Sloane, 1977). This classification is clearly of value and has important pathological and clinical implications. Tumours growing with an infiltrative growth pattern have increased local aggressiveness and tendency to recurrence after treatment, and frequently invade the deeper structures beneath the skin (Sloane, 1977; Jacobs *et al.*, 1982; Leffel *et al.*, 1991). We have previously reported that E-CD expression was preserved in cases of superficial and nodular BCC, and was reduced in most cases of infiltrative BCC, suggesting a role for E-CD in the growth pattern and local invasiveness of

Correspondence: C Gamallo, Departamento de Anatomía Patológica, Hospital La Paz, Paseo de la Castellana, 261, 28046 Madrid, Spain
Received 17 November 1994; revised 16 March 1995; accepted 22 March 1995.

BCC (Pizarro et al., 1994). In another study, preserved E-CD expression in the outer cell layers of the tumour nests was also found in five nodular BCCs (Czech et al., 1993).

In the present work, we have investigated by immunohistochemistry the expression of both P- and E-CD in a new series of BCCs of skin in order to correlate cadherin expression with the cytoarchitecture and overall growth pattern of the tumours. We have also examined cadherin expression in the epidermis overlying tumours because previous studies have demonstrated that this epidermis differs from normal epidermis with regard to some biological properties, such as keratin typing and proliferative activity (Kikuchi et al., 1993).

### Materials and methods

### *Tissue specimens*

Thirty-two primary untreated BCCs, each from a different patient, were surgically removed and the diagnosis confirmed by histopathology. There were 17 men and 15 women with a mean age of 69.3 years (range 49–89). Twenty-five lesions were located on the face and seven lesions were located on the trunk. Tumours included in this series were classified in three different patterns of growth: superficial multicentric (i.e. tumour nests attached to the undersurface epidermis, with a well-demarcating peripheral palisading, seven samples); nodular expansive (i.e. tumour nests of various shapes and sizes embedded in the dermis, with a rounded smooth outline and well-developed palisading, 14 samples); and infiltrative (i.e. small cell nests and aggregates with spiky irregular configuration and absent or poorly developed peripheral palisading, 11 samples). Keratinocytic atypia in the epidermis overlying lesions (i.e. basal and/or suprabasal keratinocytes with disturbed cellular polarity and irregular, enlarged nuclei with conspicuous, sometimes multiple, darkly stained nucleoli) was observed in 16 cases. Normal human skin was obtained from cosmetic surgery procedures. Tumour tissue and normal skin obtained from fresh specimens were embedded in optimal cryopreserving tissue (OCT) compound (Miles Laboratory, Naperville, IL, USA), snap frozen in liquid nitrogen-cooled isopentane and stored at −70°C. The remaining tumour tissue was routinely fixed in 10% formalin for 24 h and embedded in paraffin.

### *Antibodies*

Two mouse monoclonal antibodies specific for human P-CD (NCC-CAD-299) and human E-CD (HECD-1) (Shimoyama et al., 1989) were used. Both monoclonal antibodies were kindly provided by Professor M Takeichi (Department of Biophysics, Faculty of Science, Kyoto University, Japan).

### *Cadherin expression by immunohistochemical technique*

Immunostaining was performed by the streptavidin–biotin–alkaline phosphatase method as previously reported (Gamallo et al., 1993; Pizarro et al., 1994), with some modifications. Briefly, cryostat sections of 5–6 µm thickness were cut, air dried for 15 min, fixed in 10% formalin in Tris buffer containing 10 mM $Ca^{2+}$, pH 7.2, for 3 min, and then post-fixed in methanol at −10°C for 1 min and in acetone at 4°C for 3 min. After washing, non-specific binding was blocked with 5% non-fat milk, 0.1% Triton ×-100 (Sigma, St Louis, MO, USA) for 30 min at room temperature. The slides were incubated with the MAbs NCC-CAD-299 and HECD-1 for 1 h at 37°C in a humidified chamber. The primary antibodies were used at a dilution of 1:20 and 1:200 respectively, made in 150 mM sodium chloride, 10 mM Hepes pH 7.4, 10 mM calcium chloride (HMC-Ca buffer), containing 1% (w/v) bovine serum albumin (BSA). After washing in Tris buffer pH 7.4 with 10 mM $Ca^{2+}$, the sections were incubated with biotinylated goat anti-mouse IgG (Biomakor, Rehovot, Israel) diluted 1:200 for 30 min at 37°C, followed by a 30 min incubation with a 1:250 dilution of streptavidin–alkaline phosphatase complex (Dako, Glostrup, Denmark) at 37°C. Dilution of both the secondary antibody and the streptavidin–alkaline phosphatase complex was made in Tris buffer–1% bovine serum albumin (BSA). The alkaline phosphatase activity was developed using naphthol AS-MX phosphate (Sigma) as substrate and fast red dye (Sigma) as the chromogen group. Sections were finally counterstained with Meyer haematoxylin and mounted for light microscopic study. Negative controls consisted of sections of each tumour in which the primary antibody was replaced by an irrelevant mouse monoclonal antibody of the same isotype. Normal human skin was used as a positive control.

### *Evaluation of the immunohistochemical staining*

Staining for each cadherin was evaluated separately in both the outer and the inner cell layers of the tumour nests in each specimen in order to correlate the intensity and distribution of cadherin expression with the cytoarchitecture and growth pattern of the tumours. Preserved cadherin expression implied strong or moderate staining intensity in the majority of cells. Reduced cadherin expression was considered when most cells were weakly or negatively stained, either in the periphery or in the central part of tumour nests (partially reduced cadherin expression) or in both (globally reduced cadherin expression).

Regarding cadherin expression in the overlying epidermis, P-CD was considered to be normally expressed when it was restricted to the basal layer. The expansion of the P-CD positive cells in the spinous layer was defined as anomalous P-CD expression. On the other hand, reduced E-CD expression implied that the intensity of immunostaining in the spinous layer was weaker than that observed in normal epidermis.

### *Statistical analysis*

The chi-square test was used to analyse the statistical significance between cadherin expression in the tumours and the growth pattern, as well as between cadherin expression in the overlying epidermis and the presence of keratinocytic atypia, the location of the tumours and their growth pattern.

### Results

### *Cadherin expression in BCC*

The expression of P- and E-CD in different histological types of BCC is summarised in Table I. Both cadherins were expressed in all specimens with variable intensity and distribution, showing a linear pattern around the periphery of tumour cells. Weak cytoplasmic staining was occasionally noted.

**Table I** Cadherin expression in different histological types of BCC

| | *P-cadherin* | | *E-cadherin* | | |
|---|---|---|---|---|---|
| *Growth pattern* | *OCL* | *ICL* | *OCL* | *ICL* | *No. of cases* |
| Superficial | Pre | Pre | Pre | Pre | 5 |
| | Pre | Pre | Red | Pre | 2 |
| Nodular | Pre | Red | Red | Pre | 4 |
| | Pre | Pre | Pre | Pre | 3 |
| | Pre | Red | Pre | Pre | 3 |
| | Pre | Pre | Red | Pre | 3 |
| | Pre | Pre | Pre | Red | 1 |
| Infiltrative | Pre | Pre | Red | Red | 9 |
| | Pre | Pre | Pre | Pre | 2 |

OCL, outer cell layers; ICL, inner cell layers; Pre, preserved; Red, reduced.

Five of seven (71%) cases of superficial multicentric BCC showed preserved expression of the two cadherins in both the outer and the inner cell layers of the tumour nests (Figure 1a and b). The remaining two cases showed preserved P-CD expression throughout the tumour mass, whereas E-CD staining was preserved in the central part of the tumour nests but reduced in the outer cell layers.

Only three out of 14 (21%) cases of nodular BCC showed preserved expression of both cadherins in all the tumour cells. In the remaining 11 cases (79%) the pattern of cadherin expression was highly variable, as indicated in Table I. It is noteworthy that all nodular BCCs have preserved P-CD expression in the outer cell layers of the tumour nests. However, seven cases (50%) showed reduced P-CD expression in the inner cell layers. Eight out of 14 (57%) specimens showed reduced E-CD expression, either in the periphery (seven cases) or the central part (one case) of the tumour nests. Four cases with histopathological signs of limited follicular differentiation had a similar pattern of cadherin expression, showing preserved P-CD/reduced E-CD expression in the outer cell layers, but reduced P-CD/preserved E-CD expression in the inner cell layers (Figure 1c and d).

Preserved P-CD expression throughout the tumour mass was detected in all cases of infiltrative BCC (Figure 1e). In contrast, nine out of 11 (78%) infiltrative BCCs showed reduced E-CD staining in both the periphery and central part of the tumour nests (globally reduced E-CD expression). Among them, immunostaining was homogeneously reduced (very weak or even absent) in almost all the tumour cells in two cases (Figure 1f). In the remaining seven cases the cells showing different immunostaining intensity were intermingled in tumour nests without any definite spatial distribution, the cells with weak or negative staining being more abundant. Strong immunostaining for E-CD was focally observed in some cases in cellular aggregates showing squamous differentiation and horn pearls formation (Figure 1f). The statis-

**Figure 1** Immunostaining for P-cadherin (**a**, **c** and **e**) and E-cadherin (**b**, **d** and **f**) in different histological types of basal cell carcinoma (BCC). Superficial BCC with preserved expression of P-CD (**a**) and E-CD (**b**). Nodular BCC, with preserved expression of P-CD in the outer cell layers (**c**) and preserved expression of E-CD in the central part of tumour nests (**d**). Infiltrative BCC showing preserved expression of P-CD (**e**) and very weak or even absent E-CD immunostaining (**f**) in almost all tumour cells. Note the strong E-CD staining in a cellular aggregate with squamous differentiation and horn pearl formation (**f**, arrowheads).

tical analysis showed a significant association between the infiltrative growth pattern and globally reduced E-CD expression ($P<0.01$), as well as between partially reduced P-CD expression (always in the central part of tumour nests) and the nodular growth pattern ($P<0.01$).

*Cadherin expression in the epidermis overlying tumours*

Results are summarised in Table II. Twelve out of 32 cases (36%) showed normal expression of both cadherins in the epidermis overlying tumours. Eleven of these cases (92%) had no histopathological signs of keratinocytic atypia. Twenty out of 32 cases (64%) showed anomalous P-CD expression, which was observed in both basal and suprabasal keratinocytes (Figure 2a). Among them, ten cases had normal E-CD expression and the remaining ten cases had reduced E-CD expression (Figure 2b). Epidermis distal from the tumours had a normal pattern of cadherin expression (Figure 2c and d), and disturbed cadherin expression becomes progressively more pronounced with increasing proximity to tumour tissue. The statistical analysis showed a significant association between the presence of keratinocytic atypia and both anomalous P-CD expression ($P<0.01$) and reduced E-CD expression ($P=0.05$). In contrast, neither tumour location nor the histological type of BCC appear to be significantly associated with disturbed cadherin expression in the overlying epidermis.

**Table II** Cadherin expression in epidermis overlying tumours: correlation with the presence of keratinocytic atypia, location of the lesions and their growth pattern

| | Cadherin expression | | |
|---|---|---|---|
| | *Normal P-CD Normal E-CD* | *Anomalous P-CD Normal E-CD* | *Anomalous P-CD Reduced E-CD* |
| Keratinocytic atypia | | | |
| Absent | 11 | 3 | 2 |
| Present | 1 | 7 | 8 |
| Location | | | |
| Face | 10 | 8 | 7 |
| Trunk | 2 | 2 | 3 |
| Growth pattern | | | |
| Superficial | 3 | 3 | 1 |
| Nodular | 6 | 4 | 4 |
| Infiltrative | 3 | 3 | 5 |

### Discussion

BCC of the skin is a low-grade tumour with a low potential for metastasis but with a significant risk of local invasion, destruction and recurrence. Many factors seem to be implicated in the local aggressiveness and growth pattern of BCC, such as the integrity and composition of the basement membrane zone, the release of proteases implicated in the extracellular matrix degradation and the expression of cell-to-substrate and cell-to-cell adhesion molecules, such as integrins and cadherins (Miller, 1991*a*; Stamp and Pignatelli, 1991; Pizarro *et al*., 1994).

We have previously reported preserved E-CD expression in almost all tumour cells in cases of superficial and nodular BCC of skin (Pizarro *et al*., 1994). In the present series, we have observed that many cases of nodular BCC and some cases of superficial BCC had partially reduced E-CD expression, being composed of two populations of cells with different E-CD immunostaining. It is noteworthy that, in

**Figure 2** Immunostaining for P-cadherin (**a** and **c**) and E-cadherin (**b** and **d**) in the epidermis overlying a basal cell carcinoma (**a** and **b**) and in the normal epidermis distal to the tumour (**c** and **d**). Strong P-CD (**a**) and weak E-CD staining (**b**) is detected in the spinous layer of epidermis adjacent to tumour. In contrast, P-CD expression is restricted to the basal layer of both epidermis (**c**) and adnexal structures (**c**, arrowheads) in the skin distal to the tumours. Strong E-CD staining is observed in the spinous layer of the distal epidermis (**d**).

these tumours, cells showing a similar amount of E-CD tend to be grouped. A similar finding was also reported by Czech *et al.* (1993). Thus, a relatively complex picture seems to emerge from the various studies on E-CD expression in specimens of nodular and superficial BCC. There are three main patterns of E-CD expression in these tumours: (1) preserved expression throughout the tumour mass; (2) preserved expression in the outer cell layers and reduced expression in the inner ones; and (3) reduced expression in the outer cell layers and preserved expression in the central part of the tumour nests. In contrast, E-CD expression was reduced in most cases of infiltrative BCC, both in the periphery and in the central part of tumour nests (globally reduced E-CD expression). These cases showed either a weak or even absent E-CD immunoreactivity in most tumour cells or a mixed population of cells showing different E-CD staining intensity without any definite spatial distribution throughout the tumour mass. All these observations suggest that both the intensity and the topography of E-CD expression may be related to the overall growth pattern of the tumours and that an ordered pattern of E-CD expression, as observed in superficial and nodular BCCs, may be related to reduced invasiveness. In contrast, globally reduced E-CD expression and a disordered distribution of cells with different E-CD staining may favour the infiltrative growth pattern.

The expression of P-CD in tumour cells of BCC was expected, since P-CD is present in basal cells of both normal epidermis and hair follicles (Shimoyama *et al.*, 1989; Fujita *et al.*, 1992). P-CD expression was preserved in the outer cell layers of tumour nests in all specimens without exception. However, some cases of nodular BCC showed reduced P-CD expression in the central part of tumour nests which could be related with the state of differentiation of these cells, since P-CD expression is down-regulated in both suprabasal keratinocytes of the epidermis and the inner root sheath of embryonic hair follicles (Nose and Takeichi, 1986; Hirai *et al.*, 1989; Fujita *et al.*, 1992). Furthermore, several different patterns of P- and E-CD expression were observed in superficial and particularly in nodular BCC when we examined both cadherins together. This diversity could be the result of the pluripotential nature of the cell of origin of BCC and could be related in part to the degree of differentiation of tumour cells either towards epidermis or adnexal structures. Interestingly, four nodular BCC showing histological signs of limited follicular differentiation displayed the same pattern of cadherin expression, resembling in part that observed in hair bulbs during hair development (Fujita *et al.*, 1992; Kaplan and Holbrook, 1994).

A relevant finding of this study is that all the infiltrative tumours analysed showed preserved P-CD expression in almost all the tumour cells, which is in contrast to the reduced E-CD expression usually observed in this type of BCC. It strongly suggests that P-CD expression does not prevent local invasiveness and aggressive behaviour of BCC. In this sense, it should be pointed out that experimental observations in cell lines coexpressing both cadherins seem to support that cell–cell interactions mediated by P-CD may be more unstable than those mediated by E-CD (Wu *et al.*, 1993). A role for P-CD in less permanent cell–cell associations is also consistent with the restricted expression of this cadherin to basal proliferating cells of stratified epithelia, where their cell–cell contacts must be frequently broken and reformed (Nose and Takeichi, 1986; Shimoyama *et al.*, 1989). Thus, it is conceivable that preserved P-CD expression together with reduced E-CD expression throughout the tumour mass may reduce cohesiveness between tumour cells and may favour the infiltrative growth pattern. However, P-CD expression may contribute to the maintenance of the basaloid epithelial phenotype in tumour cells with severely reduced or even absent E-CD expression.

Particularly interesting is the finding that anomalous P-CD expression was frequently observed in the spinous layer of epidermis overlying tumours. In addition, some of these cases also showed reduced E-CD expression. This is in line with previous observations showing several biological abnormalities, such as increased proliferative activity and abnormal expression of keratins, involucrin, transforming growth factor beta and epidermal growth factor receptor, in the epidermis adjacent to BCC (Said *et al.*, 1984; Asada *et al.*, 1993; Kikuchi *et al.*, 1993; Stamp *et al.*, 1993). It has been suggested that diffusible factors elaborated by malignant cells and/or activated dermal cells could interfere with the normal differentiation of adjacent normal cells, causing altered expression of several antigens in non-neoplastic keratinocytes adjacent to tumours (Wolf and Bystryn, 1981). The inflammatory response to the tumour tissue or to ulceration could play some role in this phenomenon. Thus, it would be of interest to investigate further whether cytokines and growth factors modulate cadherin expression in epidermal keratinocytes. Alternatively, abnormalities in the overlying epidermis may reflect field cancerisation of the skin (Kikuchi *et al.*, 1993). In fact, our observation that disturbed cadherin expression was significantly associated with the presence of keratinocytic atypia suggests that it could be an early indication of malignant transformation.

In summary, our results suggest that the patterns of expression of P- and E-CD are related, at least in part, with the cytoarchitecture of BCC, and that P- and E-CD must play different roles in the growth pattern and local invasiveness of BCC. In particular, preserved P-CD expression together with severely reduced E-CD expression throughout the tumour mass may favour the locally aggressive behaviour of BCC. In addition, disturbed cadherin expression is frequently found in the overlying epidermis and could be a biological marker for a hyperproliferative state and/or premalignant changes in human epidermis.

**Acknowledgements**

We thank Inmaculada Briones and Petra Rubio for technical assistance with the immunohistochemical study. We are also grateful to Professor M Takeichi for his generous gift of the MAbs NCC-CAD-299 and HECD-1. This work was supported by the Fondo de Investigación Sanitaria (FIS 92/0505 and 95/0849). A Pizarro was recipient of a grant (BAE 94/5027) from the Fondo de Investigación Sanitaria, Spain.

### References

ASADA M, SCHAART FM, DE ALMEIDA HL, KORGE B, KUROKAWA I, ASADA Y AND ORFANOS CE. (1993). Solid basal cell epithelioma (BCE) possibly originates from the outer root sheath of the hair follicle. *Acta Derm. Venereol. (Stockh.)*, **73,** 286–292.

BIRCHMEIER W AND BEHRENS J. (1994). Cadherin expression in carcinomas: role in the formation of cell junctions and the prevention of invasiveness. *Biochim. Biophys. Acta*, **1198,** 11–26.

CZECH W, KRUTMANN J, HERRENKNECHT K, SCHOPF E AND KAPP A. (1993). Human cell adhesion molecule uvomorulin is differentially expressed in various skin tumors. *J. Cutan. Pathol.*, **20,** 168–172.

FUJITA M, FURUKAWA F, FUJII K, HORIGUCHI Y, TAKEICHI M AND IMAMURA S. (1992). Expression of cadherin cell adhesion molecules during human skin development: morphogenesis of epidermis, hair follicles and eccrine sweat ducts. *Arch. Dermatol. Res.*, **284,** 159–166.

GAMALLO C, PALACIOS J, SUAREZ A, PIZARRO A, NAVARRO P, QUINTANILLA M AND CANO A. (1993). Correlation of E-cadherin expression with differentiation grade and histological type in breast carcinoma. *Am. J. Pathol.*, **142,** 987–993.

GRIMWOOD RE, SIEGLE RJ, FERRIS CF AND HUFF JC. (1986). The biology of basal cell carcinoma. A revisit and recent developments. *J. Dermatol. Surg. Oncol.*, **12**, 805-808.

GRUNWALD GB. (1993). The structural and functional analysis of cadherin calcium-dependent cell adhesion molecules. *Curr. Opin. Cell Biol.*, **5**, 797-805.

HIRAI Y, NOSE A, KOBAYASHI S AND TAKEICHI M. (1989). Expression and role of E- and P-cadherin adhesion molecules in embryonic histogenesis. II. Skin morphogenesis. *Development*, **105**, 271-277.

JACOBS GH, RIPPEY JJ AND ALTINI MV. (1982). Prediction of aggressive behavior in basal cell carcinoma. *Cancer*, **49**, 533-537.

JOHNSON KR, LEWIS JE, LI D, WAHL J, PERALTA-SOLER A, KNUDSEN KA AND WHEELOCK MJ. (1993). P- and E-cadherin are in separate complexes in cells expressing both cadherins. *Exp. Cell Res.*, **207**, 252-260.

KAPLAN ED AND HOLBROOK KA. (1994). Dynamic expression patterns of tenascin, proteoglycans, and cell adhesion molecules during human hair follicle morphogenesis. *Dev. Dyn.*, **199**, 141-155.

KIKUCHI A, SAKURAOKA K, SHIMIZU H AND NISHIKAWA T. (1993). Immunohistochemical evaluation of epidermis overlying basal cell carcinomas. *Br. J. Dermatol.*, **128**, 644-649.

LEFFEL DJ, HEADINGTON JT, WONG DS AND SWANSON NA. (1991). Aggressive-growth basal cell carcinoma in young adults. *Arch. Dermatol.*, **127**, 1663-1667.

LEVER WF AND SCHAUMBURG-LEVER G. (1990). *Histopathology of the Skin*, 7th edn. JB Lippincott: Philadelphia.

LEWIS JE, JENSEN PJ AND WHEELOCK MJ. (1994). Cadherin function is required for human keratinocytes to assemble desmosomes and stratify in response to calcium. *J. Invest. Dermatol.*, **102**, 870-877.

MAREEL M, BRACKE M AND VAN ROY F. (1994). Invasion promoter versus invasion suppressor molecules: the paradigm of E-cadherin. *Mol. Biol. Rep.*, **19**, 45-67.

MILLER SJ. (1991*a*). Biology of basal cell carcinoma (Part I). *J. Am. Acad. Dermatol.*, **24**, 1-13.

MILLER SJ. (1991*b*). Biology of basal cell carcinoma (Part II). *J. Am. Acad. Dermatol.*, **24**, 161-175.

NAVARRO P, GOMEZ M, PIZARRO A, GAMALLO C, QUINTANILLA M AND CANO A. (1991). A role for the E-cadherin cell-cell adhesion molecule during tumor progression of mouse epidermal carcinogenesis. *J. Cell Biol.*, **115**, 517-533.

NOSE A AND TAKEICHI M. (1986). A novel cadherin cell adhesion molecule: its expression patterns associated with implantation and organogenesis of mouse embryos. *J. Cell Biol.*, **103**, 2649-2658.

PIZARRO A, BENITO N, NAVARRO P, PALACIOS J, CANO A, QUINTANILLA M, CONTRERAS F AND GAMALLO C. (1994). E-cadherin expression in basal cell carcinoma. *Br. J. Cancer*, **69**, 157-162.

RASBRIDGE SA, GUILLETT CE, SAMPSON SA, WALSH FS AND MILLIS RR. (1993). Epithelial (E-) and placental (P-) cadherin cell adhesion molecule expression in breast carcinoma. *J. Pathol.*, **169**, 245-250.

SAID JW, SASSOON AF, SHINTAKU IP AND BANKS-SCHLEGEL S. (1984). Involucrin in squamous and basal cell carcinomas of the skin. An immunohistochemical study. *J. Invest. Dermatol.*, **82**, 449-452.

SAKAKI T, WATO M, KAJI R, MUSHIMOTO K, SHIRASU R AND TANAKA A. (1994). Correlation of E- and P-cadherin expression with differentiation grade and mode of invasion in gingival carcinoma. *Pathol. Int.*, **44**, 280-286.

SHIMOYAMA Y AND HIROHASHI S. (1991). Expression of E- and P-cadherin in gastric carcinomas. *Cancer Res.*, **51**, 2185-2192.

SHIMOYAMA Y, HIROHASHI S, HIRANO S, NOGUCHI M, SHIMOSATO Y, TAKEICHI M AND ABE O. (1989). Cadherin cell adhesion molecules in human epithelial tissues and carcinomas. *Cancer Res.*, **49**, 2128-2133.

SLOANE JP. (1977). The value of typing basal cell carcinomas in predicting recurrence after surgical excision. *Br. J. Dermatol.*, **96**, 127-132.

STAMP GWH AND PIGNATELLI M. (1991). Distribution of beta-1, alpha-1, alpha-2 and alpha-3 integrin chains in basal cell carcinoma. *J. Pathol.*, **163**, 307-313.

STAMP GWH, NASIM M, CARDILLO M, SUDHINDRA SG, LALANI EN AND PIGNATELLI M. (1993). Transforming growth factor-beta distribution in basal cell carcinoma: relationship to proliferation index. *Br. J. Dermatol.*, **129**, 57-64.

TAKEICHI M. (1991). Cadherin cell adhesion receptors as a morphogenetic regulator. *Science*, **251**, 1451-1455.

TAKEICHI M. (1993). Cadherins in cancer. Implications for invasion and metastasis. *Curr. Opin. Cell Biol.*, **5**, 806-811.

WADE TR AND ACKERMAN AB. (1978). The many faces of basal cell carcinoma. *J. Dermatol. Surg. Oncol.*, **4**, 23-28.

WHEELOCK MJ AND JENSEN PJ. (1992). Regulation of keratinocyte intercellular junction organization and epidermal morphogenesis by E-cadherin. *J. Cell Biol.*, **117**, 415-425.

WOLF D AND BYSTRYN JC. (1981). Alterations in antigenic properties of normal epidermis adjacent to basal cell carcinomas. *J. Invest. Dermatol.*, **76**, 442-444.

WU JC, GREGORY CW AND DEPHILIP RM. (1993). P-cadherin and E-cadherin are coexpressed in MDCK cells. *Biochem. Biophys. Res. Commun.*, **195**, 1329-1335.

YASUI W, SANO T, NISHIMURA K, KITADAI Y, JI ZQ, YOKOZAKI H, ITO H AND TAHARA E. (1993). Expression of P-cadherin in gastric carcinoma and its reduction with tumor progression. *Int. J. Cancer*, **54**, 49-52.